# Flow behavior, thixotropy, and dynamic viscoelasticity of ethanolic purified basil (*Ocimum bacilicum* L.) seed gum solutions during thermal treatment

**DOI:** 10.1002/fsn3.992

**Published:** 2019-04-11

**Authors:** Reza Farahmandfar, Mohammad Reza Salahi, Maryam Asnaashari

**Affiliations:** ^1^ Department of Food Science & Technology Sari Agricultural Sciences & Natural Resources University (SANRU) Sari Iran; ^2^ Department of Food Science & Technology Ferdowsi University of Mashhad (FUM) Mashhad Iran

**Keywords:** Cox–Merz rule, dynamic modulus, purified basil seed gum, shear dependency, time dependency

## Abstract

During processing, foodstuffs may be treated at various thermal operations. Thus, this study investigated the functional properties and flow behavior at operation conditions to ensure safety and improve quality and stability at high temperatures and analyzed the ability of gum to be used in food formulation. The results showed that the purified basil seed gum (PBSG) solutions could beknown as non‐Newtonian liquids with pseudoplastic behavior. Frequency sweep revealed the storage modulus (*G*′) was higher than the loss modulus (*G*″) in the treatments. According to stress sweep, frequency sweep, complex viscosity (*η* ^*^), and loss‐tangent (tan *δ*) outcomes, mechanical spectra of PBSG were categorized as weak gels. Besides, concentration and temperature were effect on *G*′ and *G*″. The results indicated that, in general, 1% PBSG‐121°C had the maximum yield stress, consistency coefficient (*k*), extent of thixotropy, and the minimum values of flow behavior index. Also, 1% PBSG showed the highest *G*′, *G*″, *η* ^*^, yield stress values at the limit of the LVE range (τ_y_), flow‐point stress (τ_f_), and corresponding modulus *G*_f_ (*G*′ = *G*″), and the lowest value of tan *δ*. Exhibiting distinctive rheological characteristics of PBSG makes it as a worthy hydrocolloid to use in food products, which use thermal processing.

## 1 INTRODUCTION

Basil seed gum (BSG) is an original hydrocolloid with pseudoplastic behavior that can be extracted from *Ocimum basilicum* L. BSG has high intrinsic viscosity (39.17 dl/g) and molecular weight (2,320 kg/mol), which generally comprises of two different molecular weight fractions: SUPER‐BSG (6,000 kg/mol) and PER‐BSG (1,045 kg/mol) (Naji‐Tabasi, Razavi, Mohebbi, & Malaekeh‐Nikouei, 2016). The polysaccharides extracted from basil seed comprise of 43% glucomannan as the hydrophobic fraction, 24.29% of xylan with hydrophilic properties, and 2.31% of glucan (Hosseini‐Parvar, Matia‐Merino, Goh, Razavi, & Mortazavi, 2010). BSG due to bioavailability and biodegradability as well as high potential of emulsifying, thickening, gelling, filming, and stabilizing agent has achieved a great attention in the food industry and pharmacy (Naji‐Tabasi & Razavi, 2017a). Awareness of the rheological characteristics is important in the design of food processing and modeling. Therefore, understanding rheological properties of BSG is necessary for proper use of this hydrocolloid functionality in various food systems.

During processing, foodstuffs may be treated at various thermal operations like sterilization, pasteurization, baking, cooking, and drying. Therefore, this study investigated the functional properties and flow behavior at operation conditions to ensure safety and improve quality and stability at high temperatures and analyzed the ability of gum to be used in food formulation (Naji, Razavi, & Karazhiyan, 2012; Zameni, Kashaninejad, Aalami, & Salehi, 2015). Results showed that in dilute regime, BSG has random coil conformation, but can form ordered conformation at desirable situations like appropriate concentration, existence of binding agents, or alteration in temperature and pH (Naji‐Tabasi et al., 2016).

Several studies have been performed to use BSG as a new hydrocolloid in the food products (Farahmandfar, Asnaashari, Salahi, & Rad, 2017; Hosseini‐Parvar, Matia‐Merino, & Golding, 2015; Hosseini‐Parvar, Osano, & Matia‐Merino, 2016; Naji‐Tabasi & Razavi, 2017b; Osano, Hosseini‐Parvar, Matia‐Merino, & Golding, 2014; Rafe & Razavi, 2013; Rafe, Razavi, & Farhoosh, 2013; Rafe, Razavi, & Khan, 2012; Razavi, Shamsaei, Ataye, & Emadzadeh, 2012). But, no research up to the present time has been published on the broad evaluation of the steady and dynamic rheological parameters of PBSG. Thus, the chief goal of this work was to broadly investigate the steady and dynamic rheological parameters of PBSG and their dependency to temperature and concentration. Herein, the influence of concentration and temperature on the several rheological parameters obtained from shear dependency, time dependency, stress sweep, and frequency sweep tests in the linear viscoelastic (LVE) range was investigated. Finally, the ability to run the Cox–Merz relationship between complex and apparent viscosity was evaluated.

## 2 MATERIALS AND METHODS

### 2.1 Materials

Seeds of basil (*O. bacilicum* L.) were purchased from a local market, Sari, Iran. After removing the foreign bodies and matters, seeds were sealed in bags and stored in a dry and cool place before extraction operation. Ethanol (96%) and sodium azide (NaN_3_) were provided from Merck Chemical Co. (Darmstadt, Germany) and Applichem Inc. (Darmstadt, Germany), respectively.

### 2.2 BSG extraction and purification

Basil seed gum was extracted and purified as stated by Naji‐Tabasi et al. (2016), in the conditions: soaking time: 20 min, temperature: 68°C, pH: 7, and water/seed ratio: 20:1. Separation of mucilage from the swollen seeds was achieved by scraping technique. The seeds were passed through an extractor equipped with a rotating plate that scraped the mucilage layer on the seed surface. After filtration of the crude extract, the one volume of the extracted gum solution was mixed with three volumes of 96% ethanol and left overnight at 4°C to precipitate polysaccharide. The precipitate was then recovered using a sieve to allow excess solvent to drain. The final precipitate was dispersed in distilled water with continuous stirring for 25 min. The extracted gum was then dried in a dehydrator at 38°C, ground, and packed for further use.

### 2.3 Solution preparation

Hydrocolloid solutions (w/w) were prepared by dissolving a suitable amount of the PBSG powder in deionized water, which contains 0.02% (w/w) NaN_3_ as antimicrobial preservative, and stirring with a magnetic stirrer to obtain a uniform solution with concentrations of 0.5%, 0.75%, and 1%. The solutions were shaken by a roll mixer for overnight to hydrate thoroughly.

### 2.4 Thermal treatments

To determine heat stability of PBSG, steady and dynamic shear rheological properties of 0.5%, 0.75%, and 1% PBSG were evaluated after heat treatment of the solutions at four levels including 20 (witness sample), 60, and 100°C for 30 min as well as at 121°C for 15 min (sterilization treatment) according to Hosseini‐Parvar et al. (2010). All treatments were prepared in water bath (Memmert, Schwabach, Germany), except 121°C, which was performed in an autoclave (Famco, Tehran, Iran). Before experiments, solutions were cooled to 20°C.

### 2.5 Rheological evaluations

Rheological measurement of solutions was carried out in a controlled stress/strain rheometer (Physica MCR 301, Anton Paar GmbH, Stuttgart, Germany) equipped with cone–plate system (0.208 mm gap, 50 mm diameter, and 2° angle) with at least duplication measurements. Before experiment, solutions were authorized to equilibrate to 20°C for 15 min. The temperature‐controlled system was a Peltier system (Viscotherm VT2) fully equipped with a fluid circulator (Anton Paar, GmbH) with the accuracy ± 0.01°C. Data were analyzed by the Rheoplus software version 3.40 for analysis.

#### 2.5.1 Steady shear measurements

##### Shear dependence

Due to the time‐independent quality of the gum solutions, primarily, a constant shear rate (100 s^−1^) was used until an equilibrium state was perceived. At that time, the steady shear flow properties of prepared PBSG solutions were quantified at 20°C in shear rates of 0.1–300 s^−1^. Shear stress (*τ*)–shear rate ($\dot{\gamma }$) data were fitted with the power law (Equation (1)), Carreau (Equation (2)), and Herschel–Bulkley (Equation (3)) models (Silva, Gonçalves, & Rao, 1992; Steffe, 1996):

(1) $$\tau =k_{P}{\left(\dot{\gamma }\right)}^{n_{P}}$$

(2) $$\eta =\eta_{\infty }+\frac{\eta_{0}-\eta_{\infty }}{{\left(1+{\left(\lambda \dot{\gamma }\right)}^{2}\right)}^{N}}$$

(3) $$\tau =k_{H}{\left(\dot{\gamma }\right)}^{n_{H}}+\tau_{0H}$$

where *k* and *n* are the consistency coefficient (Pa.s*^n^*) and flow behavior index (dimensionless), respectively. *τ*_0H_ is the yield stress (Pa), *η*_0_ and *η*_∞_ are the viscosity at zero and infinite shear rate, respectively (Pa.s), *N* is dimensionless exponent, and *λ* is relaxation time (s).

##### Time dependence

To study time‐dependent rheological behavior, the PBSG solutions were sheared at effective oral shear rate constant (50 s^−1^) (Bourne, 2002) and the *τ* and the viscosity (*η*) were measured as a function of shearing time (*t*) until a balance mode was achieved. The time‐dependent flow patterns of the solutions were analyzed by follow models:

Weltman model:

(4) τ=A+B(lnt)

First‐order stress decay, with a nonzero stress value:

(5) $$\tau -\tau_{\text{eq}}=\left(\tau_{0}-\tau_{\text{eq}}\right)\;e^{-kt}$$

Structural kinetic model (SKM):

(6) $${\left[\frac{\left(\eta -\eta_{\infty }^{{}^{\prime }}\right)}{\left(\eta_{0}^{{}^{\prime }}-\eta_{\infty }^{{}^{\prime }}\right)}\right]}^{1-n}=\left(n-1\right)\;kt+1$$

where *A* and *B* characterize the time‐dependent behavior, *k* is the breakdown rate constant, *τ*_0_ is the initial shear stress, *τ*_eq_ is the equilibrium stress, $\eta_{0}^{{}^{\prime }}$ is the initial apparent viscosity at *t* = 0, $\eta_{\infty }^{{}^{\prime }}$ is the steady‐state apparent viscosity at *t*→∞, and *n* is the order of the structural breakdown reaction. Herein, the second‐order (*n* = 2) was used to explain the SKM of PBSG solutions.

#### 2.5.2 Dynamic shear tests

##### Stress sweep

Before making detailed dynamic measurements, the linear viscoelastic (LVE) region for PBSG solutions was established by performing an amplitude sweep evaluation at controlled shear stress (CSS) state (0.006–40 Pa) at constant frequency of 1 Hz and 20°C. Data were analyzed by Physica Rheometer software (Rheoplus/32, version 3.40) and used to compute the LVE parameters of PBSG solutions (storage modulus $G_{\left(\text{LVE}\right)}^{{}^{\prime }}$; loss modulus $G_{\left(\text{LVE}\right)}^{{}^{\prime \prime }}$; loss‐tangent value (tan *δ*_LVE_); yield stress at the limit of the LVE range (*τ*_y_); flow‐point stress (*τ*_f_); and corresponding modulus *G*_f_ (*G*′ = *G*″).

##### Frequency sweep

The frequency sweep measurements were performed at 0.07 Pa (as constant stress in the LVE region), frequency range of 0.1–10 Hz, and 20°C. The mechanical spectra obtained were illustrated by the storage modulus (*G*′), loss modulus (*G*″), loss tangent (tan *δ*), complex viscosity (*η* ^*^), and slope of *η* ^*^ (*η* ^*^−*f*).

##### Correlation between dynamic and steady shear properties

Cox and Merz (1959) exhibited an empirical relevance among dynamic viscosity and shear viscosity:

(7) $$\eta^{*}\left(\omega \right)=\eta \;\left(\dot{\gamma }\right){|}_{\omega =\dot{\gamma }}$$

Based on this law, if polymer solution is devoid of energetic interactions, *η* and *η* ^*^ are equal at same values of *ω* and $\dot{\gamma }$. Herein, the Cox–Merz rule was investigated in the shear rate of 0.1–300 s^−1^ and frequency of 0.1–10 Hz.

### 2.6 Data analysis

The data were subjected to one‐way analysis of variance (ANOVA) at 95% significance level (*p* < 0.05), and Minitab 18 (Minitab Inc., Minneapolis, USA) was used to compare the means by Tukey test. Data fitting was performed by MathWorks’ MATLAB (R2016a) software, using the curve fitting toolbox.

## 3 RESULTS AND DISCUSSION

### 3.1 Steady shear rheological properties

#### 3.1.1 Time‐independent rheological properties

**Figure 1:**
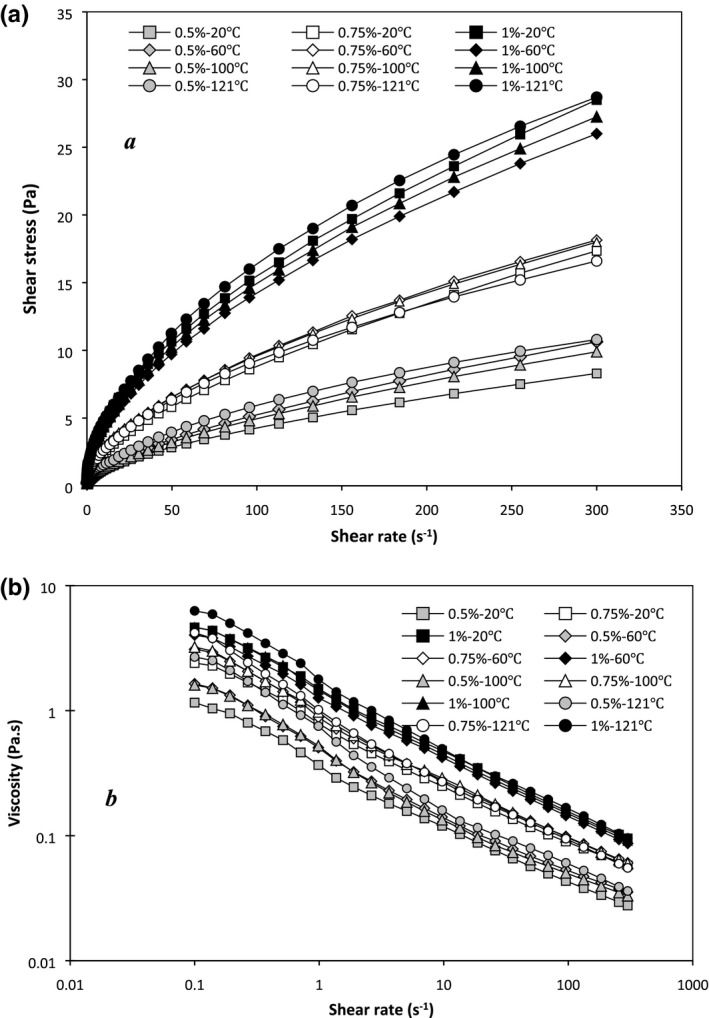
Shear stress (a) and viscosity (b) of PBSG solutions as a function of shear rate

Flow curves of shear stress and apparent viscosity as the function of shear rate of the PBSG solutions at different treatments are shown in Figure 1. The pattern of these curves and power law and Herschel–Bulkley parameters (Table 1) showed that PBSG solutions could be known as non‐Newtonian liquids with pseudoplastic behavior (*n* < 1). Vardhanabhuti and Ikeda (2006) stated that shear‐thinning demeanor of BSG is due to aggregation of polymers by hydrogen bonds and high molecular weight. As shown in Table 1, power law model exhibited high *R* ^2^ (0.9947–0.9998) and low RMSE (0.0566–0.2456). Also, *n*_p_ varied from 0.517 (1%‐121°C) to 0.602 (0.5%‐60°C). At the same temperature, with increasing PBSG concentration, generally, the *n*_p_ values were decreased that represents stronger shear‐thinning behavior. Hydrocolloids with high pseudoplastic behavior are widely used to modify or ameliorate food texture during high‐shear processing like filling and pumping, and during swallowing impart a thinner consistency (Vardhanabhuti & Ikeda, 2006). Moreover, high viscosity provides pleasant mouthfeel (Naji‐Tabasi & Razavi, 2017b). According to Table 1, the effect of thermal treatment on the *n*_p_ values was significant (*p* < 0.05). At the concentration of 0.75%, the *n*_p_ values decreased with increasing temperature, but in 0.5% and 1% BSG solutions, firstly, with increase in temperature from 20 to 60°C, *n*_p_ values were increased and then, from 60 to 121°C, were decreased.

**Table 1:** The Power law, Herschel‐Bulkley and Carreau models parameters determined for the PBSG solutions

	Power law				Herschel–Bulkley					Carreau					
	$k_{p}\left(\text{Pa}.s^{n}\right)$	*n*_p_	*R* ^2^	RMSE	*τ*_0H_ (Pa)	$k_{H}\left(\text{Pa}.s^{n}\right)$	*n*_H_	*R* ^2^	RMSE	*η*_0_(Pa.s)	*η*_∞_(Pa.s)	*λ*(*s*)	*N*(−)	*R* ^2^	RMSE
0.5%PBSG															
20	0.301 ± 0.040^g^	0.579 ± 0.003^b^	0.9993	0.0566	0.100 ± 0.027^c^	0.259 ± 0.029^e^	0.604 ± 0.007^bc^	0.9998	0.0265	1.321 ± 0.255^g^	0.032 ± 0.007^c^	6.930 ± 2.210^a^	0.347 ± 0.053^a^	0.9984	0.0134
60	0.335 ± 0.019^fg^	0.602 ± 0.005^a^	0.9981	0.1180	0.211 ± 0.028^abc^	0.255 ± 0.010^e^	0.649 ± 0.008^a^	0.9998	0.0395	1.948 ± 0.011^fg^	0.038 ± 0.002^bc^	7.747 ± 0.809^a^	0.349 ± 0.001^a^	0.9994	0.0121
100	0.325 ± 0.021^g^	0.595 ± 0.008^ab^	0.9976	0.1240	0.221 ± 0.029^abc^	0.240 ± 0.012^e^	0.647±0.010^a^	0.9997	0.0431	1.838 ± 0.054^fg^	0.031 ± 0.003^c^	6.790 ± 0.425^a^	0.349 ± 0.027^a^	0.9994	0.0114
121	0.501 ± 0.035^ef^	0.544 ± 0.004^cd^	0.9947	0.2102	0.381 ± 0.126^ab^	0.330 ± 0.019^e^	0.616 ± 0.018^ab^	0.9991	0.0905	3.393 ± 0.168^def^	0.042 ± 0.004^bc^	9.690 ± 2.550^a^	0.455 ± 0.109^a^	0.9978	0.0537
0.75%PBSG															
20	0.619 ± 0.047^de^	0.581 ± 0.007^ab^	0.9989	0.1469	0.261 ± 0.020^abc^	0.510 ± 0.036^d^	0.614 ± 0.006^ab^	0.9998	0.0661	2.773 ± 0.337^efg^	0.055 ± 0.004^abc^	7.250 ± 0.274^a^	0.319 ± 0.008^a^	0.9993	0.0189
60	0.745 ± 0.004^cd^	0.557 ± 0.002^c^	0.9993	0.1225	0.238 ± 0.005^abc^	0.640 ± 0.001^cd^	0.584 ± 0.002^bcd^	0.9999	0.0421	3.706 ± 0.105^cdef^	0.077 ± 0.009^abc^	7.802 ± 0.420^a^	0.354 ± 0.019^a^	0.9990	0.0284
100	0.791 ± 0.033^c^	0.545 ± 0.003^cd^	0.9992	0.1303	0.247 ± 0.003^abc^	0.673 ± 0.030^c^	0.573 ± 0.003^cde^	0.9998	0.0580	4.158 ± 0.624^cde^	0.060 ± 0.005^abc^	10.190 ± 1.710^a^	0.326 ± 0.004^a^	0.9992	0.0269
121	0.811 ± 0.039^c^	0.528 ± 0.007^de^	0.9986	0.1608	0.309 ± 0.020^abc^	0.658 ± 0.045^c^	0.564 ± 0.011^de^	0.9998	0.0721	6.470 ± 1.125^ab^	0.069 ± 0.017^abc^	10.062 ± 0.549^a^	0.366 ± 0.032^a^	0.9987	0.0426
1%PBSG															
20	1.357 ± 0.030^ab^	0.531 ± 0.001^de^	0.9996	0.1448	0.258 ± 0.006^abc^	1.227 ± 0.032^ab^	0.548 ± 0.002^de^	0.9999	0.0859	5.578 ± 0.389^bc^	0.085 ± 0.023^abc^	8.623 ± 0.270^a^	0.309 ± 0.020^a^	0.9993	0.0370
60	1.193 ± 0.083^b^	0.540 ± 0.006^cd^	0.9998	0.0978	0.183 ± 0.002^bc^	1.103 ± 0.081^b^	0.553 ± 0.006^de^	0.9999	0.0500	4.928 ± 0.282^bcd^	0.084 ± 0.004^abc^	9.076 ± 0.585^a^	0.309 ± 0.005^a^	0.9999	0.0370
100	1.283 ± 0.031^b^	0.534 ± 0.008^de^	0.9995	0.1566	0.292 ± 0.102^abc^	1.138 ± 0.015^b^	0.555 ± 0.014^de^	0.9999	0.0772	5.546 ± 0.423^bc^	0.092 ± 0.034^ab^	7.940 ± 1.900^a^	0.330 ± 0.039^a^	0.9989	0.0433
121	1.510 ± 0.071^a^	0.517 ± 0.004^e^	0.9990	0.2456	0.439 ± 0.111^a^	1.281 ± 0.016^a^	0.545 ± 0.009^e^	0.9996	0.1434	7.703 ± 0.673^a^	0.110 ± 0.012^a^	8.110 ± 0.898^a^	0.354 ± 0.017^a^	0.9991	0.0570

Note

Different letters indicate significant differences between PBSG solutions at *p* < 0.05 by Tukey test. [Correction added on 3 May 2019, after first online publication: Tables 1 and 2 format have been changed.]

The consistency coefficient (*k*_p_), which is related to viscosity, of power law model varied from 0.301 Pa.s*^n^* (0.5%‐20°C) to 1.510 Pa.s*^n^* (1%‐121°C). At the same temperature, *k*_p_ increased significantly (*p* < 0.05) with concentration enhancement. At the concentration of 0.75%, the *k*_p_ values increased with increasing temperature, but in 0.5% and 1% concentrations, heat treatment did not have a distinct effect on *k*_p_. However, at the same concentration, the highest *k*_p_ values were observed at 121°C, which was in agreement with the results reported by Zameni et al. (2015) and Naji‐Tabasi et al. (2016) for BSG and cress seed gum, respectively. These results illustrated that the thermal treatment creates an irreversible intermolecular arrangement in BSG that would participate to raise the viscosity.

Table 1 shows that Herschel–Bulkley model specified high *R* ^2^ (0.9991–0.9999) and low RMSE (0.0265–0.1434). Herschel–Bulkley model presented a small yield stress (*τ*_0H_) in PBSG, which is a notable parameter when gum is used as binders. The maximum and minimum *τ*_0H_ were obtained for 1%‐121°C (0.439 Pa) and 0.5%‐20°C (0.1 Pa), respectively. The results obtained for *k*_H_ and *n*_H_ parameters of the Herschel–Bulkley model were as almost the same as the results determined by the power law model, but lower and higher values were observed for *k*_H_ (0.240–1.281 Pa.s*^n^*) and *n*_H_ (0.545–0.649) in comparison with *k*_p_ and *n*_p_, respectively. Random coil conformation of BSG was stated by Naji‐Tabasi et al. (2016). Polysaccharides with random coil conformation have two Newtonian regions at low and high shear rates that called zero‐shear viscosity (*η*_0_) and infinite‐shear viscosity (*η*_∞_). The apparent viscosity of the PBSG solutions was investigated by the Carreau model, and its constant parameters are displayed in Table 1. This model is well fitted to flow curves with high *R* ^2^ (0.9978–0.9999) and low RMSE (0.0114–0.0570). The *η*_0_ and *η*_∞_ varied from 1.321 Pa.s (0.5%‐20°C) to 7.703 Pa.s (1%‐121°C) and 0.031 Pa.s (0.5%‐100°C) to 0.110 Pa.s (1%‐121°C), respectively. Under the same temperature, *η*_0_ and *η*_∞_ increased significantly (*p* < 0.05) with increasing concentration. At the same gum concentration, generally, the *η*_0_ and *η*_∞_ values increased with increasing temperature (similar trend observed for *k*_P_ and *k*_H_, as presented in Table 1). But different treatments did not have a significant effect (*p* > 0.05) on *λ* and *N* of PBSG solutions.

#### 3.1.2 Time‐dependent rheological properties

**Figure 2:**
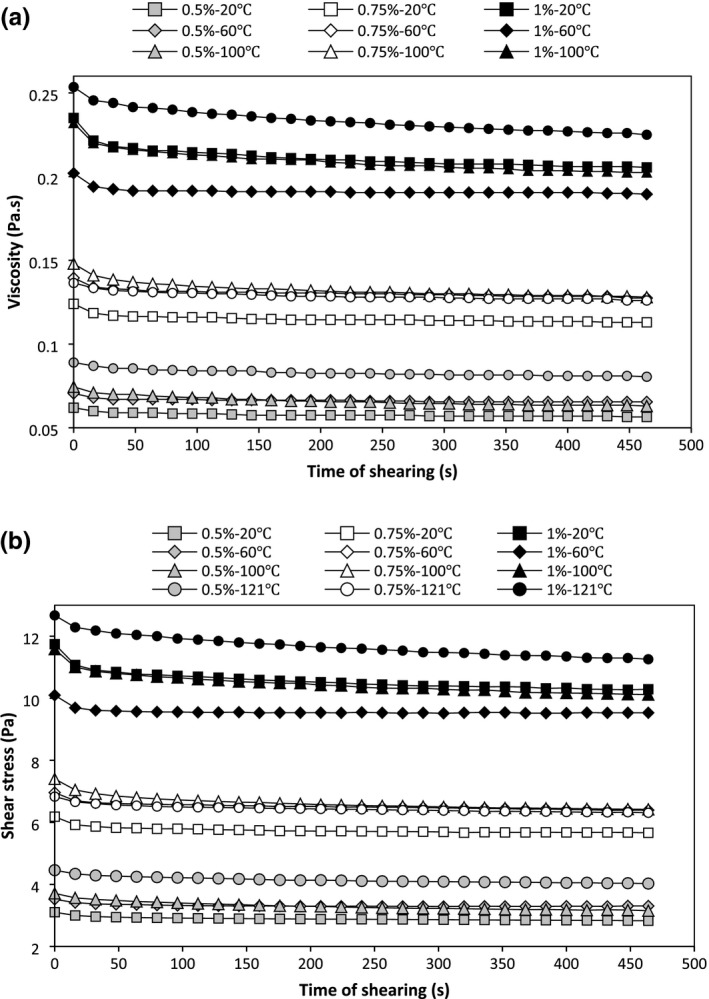
Viscosity (a) and shear stress (b) of PBSG solutions as a function of shearing time (shear rate 50 s^−1^)

Time‐dependent rheological behavior of PBSG solutions is shown in Figure 2. Results indicate that the viscosity (Figure 2a) and shear stress (Figure 2b) decreased slightly (due to low concentrations) with increasing shearing time that represents a thixotropic behavior. The thixotropic behavior of gum solutions is associated with breakdown of the network which leads to reduction of viscosity and shear stress with time. Thixotropy behavior showed that PBSG polymer chains are capable to link to each other and create a 3‐D structure. This behavior can create desirable characteristics in various food such as mayonnaise (Imeson, 2011). Three models were operated to evaluate time‐dependent structural breakdown of PBSG solutions.

##### Second‐order structural kinetic model

The SKM defaults that the variation in time‐dependent flow qualities is due to the shear‐induced breakdown of the inner structure and this breakdown rate depends on the kinetics of conversion of the structured state to the nonstructured state (Abu‐Jdayil, 2003). At a shear rate of 50 s^−1^, the apparent viscosity reduced quickly as time passed during the first 20 s and received an equilibrium state value after around 150 s (Figure 2a). This behavior showed that the arrangement of hydrocolloids is altered by the shearing force. The high *R* ^2^ (0.876–0.980) and low RMSE (0.001–0.007) values show the capacity of SKM for foretelling time‐dependent behavior of PBSG solutions (Table 2). Similar to *η*_0_ and *k*_P_, under the same temperature, $\eta_{0}^{{}^{\prime }}$ increased significantly (*p* < 0.05) with increasing concentration, and at the constant concentration, generally, the $\eta_{0}^{{}^{\prime }}$ values increased with increasing temperature. Table 2 shows that the rate constant of thixotropic breakdown, *k*, of the 1% PBSG‐60°C was significantly higher than others indicating weak connection between its chains. The extent of structural breakdown is defined by the ratio of $\eta_{0}^{{}^{\prime }}$ to $\eta_{\infty }^{{}^{\prime }}$ (Razavi & Karazhiyan, 2009). The $\eta_{0}^{{}^{\prime }}/\eta_{\infty }^{{}^{\prime }}$ obtained for 0.5% PBSG‐100°C was slightly higher than other samples indicating that the attendance of both fractions outputs in extent of thixotropy. In this condition, it could be expected to have stronger gel structure and fundamental reformation occurred (Naji‐Tabasi & Razavi, 2017b).

**Table 2:** The second‐order structural kinetics (*n* = 2), Weltman and first‐order stress decay with a non‐zero stress value models parameters determined for the PBSG solutions

	Second‐order structural kinetic					Weltman				First‐order stress decay with a non‐zero stress value				
	$\eta_{0}^{\prime }\left(\text{Pa}.s\right)$	$\eta_{0}^{\prime }/\eta_{\infty }^{\prime }$	*k* (*s* ^−1^)	*R* ^2^	RMSE	*A*(Pa)	−*B*(Pa)	*R* ^2^	RMSE	$\tau_{0}\left(\text{Pa}\right)$	$\tau_{\text{eq}}\left(\text{Pa}\right)$	$k(s^{-1})$	*R* ^2^	RMSE
0.5%PBSG														
20	0.062 ± 0.004^g^	1.101 ± 0.033^b^	0.027 ± 0.022^b^	0.941	0.002	3.024 ± 0.268^d^	0.0271 ± 0.0128^c^	0.884	0.021	3.071 ± 0.298^e^	2.845 ± 0.378^d^	0.0237 ± 0.0217^ab^	0.948	0.013
60	0.070 ± 0.002^g^	1.074 ± 0.004^b^	0.033 ± 0.028^b^	0.876	0.005	3.430 ± 0.077^d^	0.0230 ± 0.0037^c^	0.767	0.025	3.500 ± 0.135^de^	3.287 ± 0.119^d^	0.0304 ± 0.0297^ab^	0.915	0.015
100	0.073 ± 0.002^g^	1.233 ± 0.072^a^	0.009 ± 0.005^b^	0.975	0.003	3.595 ± 0.076^d^	0.0587 ± 0.0084^bc^	0.725	0.074	3.649 ± 0.113^de^	3.126 ± 0.250^d^	0.0076 ± 0.0048^b^	0.982	0.018
121	0.088 ± 0.000^f^	1.118 ± 0.039^b^	0.009 ± 0.002^b^	0.957	0.007	4.364 ± 0.024^d^	0.0440 ± 0.0142^c^	0.764	0.056	4.398 ± 0.021^d^	4.034 ± 0.164^d^	0.0075 ± 0.0029^b^	0.959	0.018
0.75%PBSG														
20	0.124 ± 0.004^e^	1.095 ± 0.001^b^	0.042 ± 0.003^b^	0.939	0.001	6.357 ± 0.781^c^	0.0514 ± 0.0016^bc^	0.939	0.026	6.106 ± 0.296^c^	5.694 ± 0.269^c^	0.0191 ± 0.0028^ab^	0.909	0.032
60	0.138 ± 0.000^d^	1.087 ± 0.007^b^	0.030 ± 0.025^b^	0.916	0.003	6.781 ± 0.129^c^	0.0542 ± 0.0084^bc^	0.885	0.039	6.841 ± 0.025^c^	6.403 ± 0.016^c^	0.0162 ± 0.0149^ab^	0.883	0.041
100	0.147 ± 0.002^d^	1.159 ± 0.010^ab^	0.018 ± 0.001^b^	0.976	0.001	7.117 ± 0.197^c^	0.1013 ± 0.0088^ab^	0.857	0.083	7.248 ± 0.197^c^	6.462 ± 0.130^c^	0.0113 ± 0.0007^ab^	0.951	0.049
121	0.136 ± 0.007^de^	1.089 ± 0.048^b^	0.026 ± 0.023^b^	0.943	0.004	6.696 ± 0.503^c^	0.0521 ± 0.0392^bc^	0.828	0.056	6.779 ± 0.476^c^	6.330 ± 0.175^c^	0.0324 ± 0.0296^ab^	0.916	0.024
1%PBSG														
20	0.233 ± 0.001^b^	1.140 ± 0.000^ab^	0.024 ± 0.002^b^	0.950	0.003	11.280 ± 0.056^a^	0.1455 ± 0.0003^a^	0.900	0.097	11.435 ± 0.092^a^	10.355 ± 0.078^ab^	0.0116 ± 0.0013^ab^	0.904	0.097
60	0.204 ± 0.007^c^	1.071 ± 0.006^b^	0.150 ± 0.003^a^	0.973	0.006	9.820 ± 0.537^b^	0.0526 ± 0.0008^bc^	0.925	0.029	10.093 ± 0.519^b^	9.537 ± 0.540^b^	0.0710 ± 0.005^a^	0.950	0.024
100	0.229 ± 0.005^b^	1.147 ± 0.031^ab^	0.013 ± 0.009^b^	0.953	0.001	11.180 ± 0.170^ab^	0.1469 ± 0.0046^a^	0.822	0.137	11.280 ± 0.311^ab^	10.092 ± 0.421^ab^	0.0084 ± 0.0055^b^	0.934	0.084
121	0.250 ± 0.008^a^	1.165 ± 0.033^ab^	0.006 ± 0.002^b^	0.980	0.005	12.390 ± 0.509^a^	0.14535 ± 0.0025^a^	0.706	0.189	12.460 ± 0.566^a^	11.135 ± 0.742^a^	0.0052 ± 0.0024^b^	0.975	0.055

Note

Different letters indicate significant differences between PBSG solutions at *p* < 0.05 by Tukey test. [Correction added on 3 May 2019, after first online publication: Tables 1 and 2 format have been changed.]

##### Weltman model

As shown in Table 2, the Weltman model shows an acceptable fit to the data (*R* ^2^: 0.706–0.939). Thixotropic breakdown coefficient (*B*) negative value indicated how shear stress drops from the primary stress value (*A*) to the steady‐state value (Abu‐Jdayil, Azzam, & Al‐Malah, 2001). The *A* and *B* parameters were altered in the range of 3.024–12.390 (Pa) and 0.0230–0.1469 (Pa), respectively (Table 3). The values of *A* and *B* were increased significantly (*p* < 0.05) with increasing concentration. Also, at 0.5% and 0.75% PBSG, different thermal treatments had not any meaningful difference (*p* > 0.05), but at the 1% solutions, the values of *A* and *B* parameters in 60°C were significantly lower than others. The highest *A* and *B* values were obtained for the 1% PBSG and thermal treatments of 20, 100, and 121°C samples, indicating higher structural breakdown rate by shearing at higher concentration. These data correspond thoroughly with the equivalent parameter acquired by the structural kinetic model, $\eta_{0}^{{}^{\prime }}/\eta_{\infty }^{{}^{\prime }}$ (Table 2), and were consistent with the results of Razavi and Karazhiyan (2009) for Balangu and Salep solutions.

**Table 3:** Linear viscoelastic parameters of PBSG solutions (stress sweep test, f = 1 Hz, 20°C)

Concentration/Temperature (°C)	$G_{(\text{LVE)}}^{\prime }\left(\text{Pa}\right)$	$G_{(\text{LVE)}}^{\prime \prime }\left(\text{Pa}\right)$	$\text{Tan}\delta_{\text{LVE}}$	$\tau_{y}\left(\text{Pa}\right)$	$\tau_{f}\left(\text{Pa}\right)$	$G_{f}\left(\text{Pa}\right)$
0.5%PBSG						
20	5.99 ± 0.65^h^	2.48 ± 0.06^i^	0.41 ± 0.03^a^	0.67 ± 0.02^ef^	1.08 ± 0.02^fg^	1.97 ± 0.16^i^
60	6.37 ± 0.38^h^	2.66 ± 0.06^hi^	0.42 ± 0.01^a^	0.60 ± 0.05^f^	0.97 ± 0.01^g^	2.45 ± 0.16^hi^
100	8.93 ± 0.15^gh^	3.18 ± 0.02^gh^	0.36 ± 0.00^abc^	0.60 ± 0.01^f^	1.07 ± 0.01^fg^	3.01 ± 0.04^hi^
121	10.70 ± 0.21^gh^	3.63 ± 0.10^g^	0.34 ± 0.00^bc^	0.90 ± 0.01^ef^	1.36 ± 0.01^ef^	3.56 ± 0.06^gh^
0.75%PBSG						
20	14.42 ± 1.00^fg^	5.61 ± 0.36^f^	0.39 ± 0.00^ab^	1.01 ± 0.02^def^	1.65 ± 0.11^e^	4.87 ± 0.21^fg^
60	18.61 ± 0.26^ef^	6.62 ± 0.12^e^	0.36 ± 0.01^abc^	1.13 ± 0.09^de^	2.11 ± 0.05^d^	6.07 ± 0.10^ef^
100	23.10 ± 0.54^de^	7.65 ± 0.01^d^	0.33 ± 0.01^bc^	1.41 ± 0.00^cd^	2.38 ± 0.02^cd^	7.21 ± 0.31^de^
121	26.56 ± 0.79^cd^	8.20 ± 0.05^d^	0.31 ± 0.01^c^	1.67 ± 0.10^c^	2.58 ± 0.11^c^	8.28 ± 0.25^d^
1%PBSG						
20	39.04 ± 2.59^b^	12.86 ± 0.20^b^	0.33 ± 0.02^bc^	2.17 ± 0.1^ab^	4.10 ± 0.01^a^	10.60 ± 0.21^c^
60	33.10 ± 2.97^bc^	11.93 ± 0.22^c^	0.36 ± 0.03^abc^	1.80 ± 0.12^bc^	3.09 ± 0.19^b^	10.55 ± 0.66^c^
100	46.12 ± 3.78^a^	14.50 ± 0.23^a^	0.31 ± 0.02^c^	1.72 ± 0.35^bc^	3.41 ± 0.19^b^	12.18 ± 0.92^b^
121	48.04 ± 0.46^a^	15.14 ± 0.26^a^	0.31 ± 0.00^c^	2.29 ± 0.07^a^	4.18 ± 0.11^a^	13.81 ± 0.30^a^

Note

Different letters indicate significant differences between PBSG solutions at *p* < 0.05 by Tukey test.

##### First‐order stress decay, with a nonzero stress value

There was a good settlement between the fitted results of first‐order stress decay model, with a nonzero stress, and shear stress data for PBSG solutions (0.883 < *R* ^2^ < 0.982) (Table 2). In general, the equilibrium stress (*τ*_eq_) and initial stress (*τ*_0_) values increased as concentration and temperature enhanced. The difference of initial to equilibrium shear stresses (*τ*_0_−*τ*_eq_) is used as a comparative index of the postponed structural breakdown or the amount of thixotropy. The value of (*τ*_0_−*τ*_eq_) increased at transition concentration from 0.5% to 1%. The decay rate constant, *k*, is an indication of how fast the hydrocolloid solution through shearing reaches the equilibrium stress value. Herein, *k* did not show a defined trend with temperature and concentration (Table 2), which is compatible with the results of Karazhiyan et al. (2009) for *Lepidium sativum* seed gum. 1% PBSG‐60°C sample had the highest *k* value (0.0710 s^−1^).

### 3.2 Dynamic rheological properties

#### 3.2.1 Stress sweep

**Figure 3:**
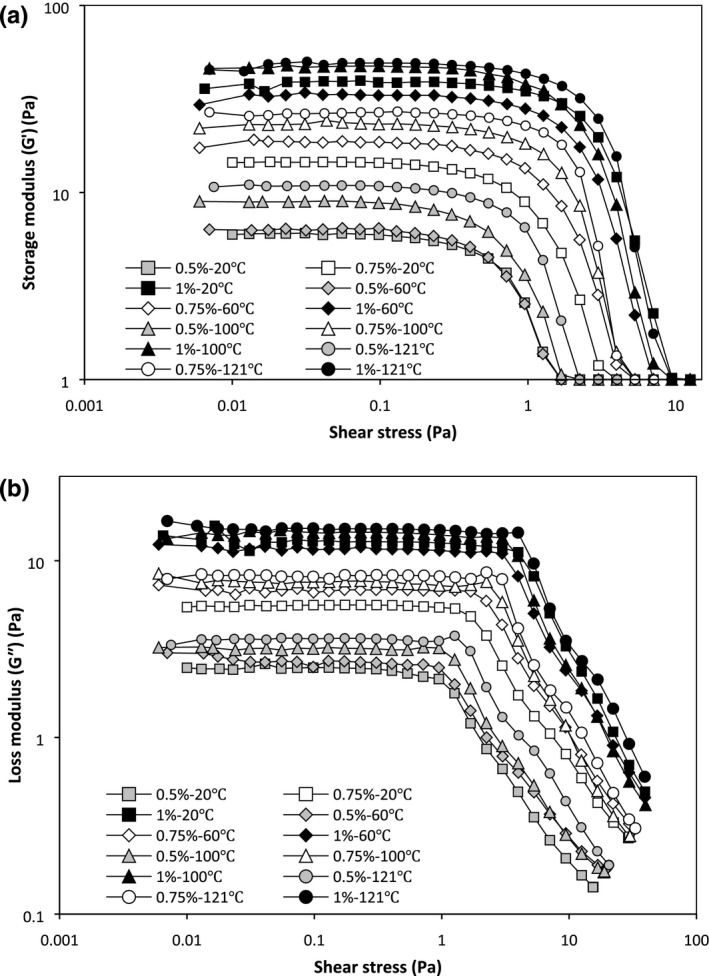
Stress sweep profile of PBSG solutions (elastic (*G*′) (a) and viscose (*G*″) (b) modulus changes) [Correction added on 3 May 2019, after first online publication: Figure 3, 4, and 5 have been replaced with the correct images.]

The amplitude sweep was carried out over the stress range 0.01–40 Pa, 1 Hz frequency, and 20°C to determine LVE region. The LVE region could be used as an indicator of gel strength, so that stronger gels in comparison with weak gels have a more extensive LVE region (Steffe, 1996). From Figure 3 and Table 3, two different regions were classified: a LVE region in which *G*′ and *G*″ were unchanging with *G*′ > *G*″ (solid‐like behavior), and a nonlinear region in which *G*′ and *G*″ start to reduce with stress enhancement. The LVE region for 0.5% PBSG in all temperatures was more limited which indicates weaker structure of them. After the crossover point, *G*″ was higher than *G*′ and the samples indicated liquid‐like behavior. Therefore, the PBSG solutions showed a gel‐like structure (a weak gel) at 1 Hz and 20°C. As shown in Figure 3a,b, at the same temperature, *G*′ and *G*″ were increased as the concentration is enhanced from 0.5% to 1%. Hence, shear stress increased with raise in PBSG concentration and reached the crossover point. At the same concentration, the storage modulus ($G_{\left(\text{LVE}\right)}^{{}^{\prime }}$) and loss modulus ($G_{\left(\text{LVE}\right)}^{{}^{\prime }}$) generally increased significantly with increasing temperature (Table 3). As shown in Figure 3 and Table 3, $G_{\left(\text{LVE}\right)}^{{}^{\prime }}$ of 1%‐100°C and 1%‐121°C solutions was higher than other samples, which reflects that these two samples have higher intermolecular interactions and entanglements.

Loss tangent (tan *δ*_LVE_) showed the ratio of *G*″ to *G*′ in each cycle. Basically, tan *δ* < 1 indicates elastic behavior and tan *δ* > 1 indicates viscous behavior. The tan *δ* > 0.1 means that the samples have a structure between real gel and high concentrated biopolymer. The tan *δ* values of PBSG solutions (0.31–0.42) were lower than 1, but higher than 0.1 (Table 3) that showed elastic structure in weak gel (Naji‐Tabasi & Razavi, 2017b). Because all PBSG solutions are not true gel, macromolecules connections and chains entanglements are provisional and could be disunited by using high shear rates. These results approved the shear‐thinning behavior of the PBSG solutions in the steady‐state experiments (See Figure 1 and flow behavior index in Table 1).

As shown in Figure 3, by increasing the shear stress, *G*′ and *G*″ started to reduce. The initial rupture (*τ*_y_) and flow point (*τ*_f_) can be assumed as dynamic yield stress (Table 3). The limiting value of LVE range in a stress sweep test is considered as yield stress or stress point (*τ*_y_). *τ*_f_ is the stress at the crossover point and shows resistance to flow. *τ*_y_ and *τ*_f_ parameters exhibited the same trend, so that, both parameters, at the same temperature, increased with increasing concentration, meaning that the gel network got stronger. At constant concentration, the highest values of *τ*_y_ and *τ*_f_ parameters were observed at 121°C. Results showed that the dynamic yield points are in agreement with steady shear yield stress (Table 1).

At the flow point, *G*′ value is considerably reduced. Therefore, the corresponding modulus (*G*_f_: *G*′ = *G*″) can be used as indicator for understanding structural strength at flow point and showed the stress at which the first nonlinear changes in the structure happened. *G*_f_ is a beneficial characteristic of gums to show their capacity to maintain the food texture (Rafe & Razavi, 2013). Treatments significantly influenced on the *G*_f_ (Table 3). At the constant temperature, *G*_f_ increased with increasing concentration, and at the same concentration, the *G*_f_ values increased with increasing temperature (the lowest *G*_f_ was for 0.5%‐20°C [1.97 Pa] and the highest *G*_f_ was for 1%‐121°C [13.81 Pa]). Results that obtained for *τ*_y_, *τ*_f_, and *G*_f_ were in agreement with the findings of Hesarinejad, Koocheki, and Razavi (2014) for *Lepidium perfoliatum* seed gum.

#### 3.2.2 Frequency sweep

**Figure 4:**
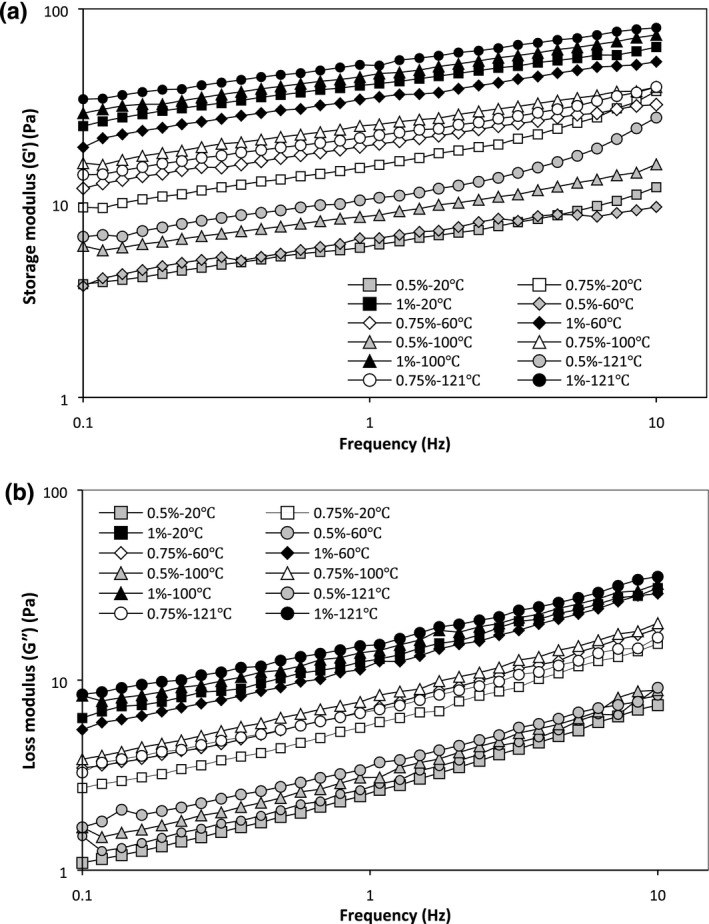
Frequency sweep profile of PBSG solutions (elastic (*G*′) (a) and viscose (*G*″) (b) modulus changes) [Correction added on 3 May 2019, after first online publication: Figure 3, 4, and 5 have been replaced with the correct images.]

The information of frequency sweep declares that dispersions could be categorized into four groups: dilute solution, concentrated solution, weak gel, and strong gel (Steffe, 1996; Vasile, 2009). For dilute solutions, *G*″ > *G*′ and is almost similar to each other at higher frequencies. In concentrated samples, *G*″ is bigger than *G*′ at low frequency and the crossover of them happens in the middle of frequency span. For gels, always *G*′ > *G*″ in frequency range, so that, strong gel is approximately independent of frequency, but in a weak gel, dynamic modulus is largely dependent on frequency (Kutz, 2013). Dynamic frequency sweep is performed in the LVE limit to specify the frequency dependence of *G*′, *G*″, complex viscosity (*η* ^*^), tan *δ*, and the slope of *η* ^*^ of the PBSG solutions. Figure 4 reveals the mechanical spectra achieved by frequency sweep test, and the rheological parameters obtained from this figure are reviewed in Table 4. Results showed that PBSG has characteristically gel‐like behavior, with *G*′ exceeding *G*″ in all frequency ranges. Moreover, in the frequency range findings, *G*′ and *G*″ did not crossover the other one. Moreover, samples with higher concentration exhibited an enhancement in *G*′ and *G*″ and a larger gap between *G*′ and *G*″ shows the ability to create macromolecular networks. Ross‐Murphy (2012) stated that rise in *G*′ and *G*″ while they are parallel together is associated with the network defects, that is to mean, in lower concentration, the high intermolecular zones could not take part in noncovalence cross‐junctions (Rincón, Muñoz, De Pinto, Alfaro, & Calero, 2009; Ross‐Murphy, 2012), but the number of junction zones created at higher concentrations (1%) is maximum. Similar observations have been reported for psyllium gel (Farahnaky, Askari, Majzoobi, & Mesbahi, 2010) and *L. perfoliatum* seed gum gel (Hesarinejad et al., 2014).

**Table 4:** Dynamic rheological parameters of PBSG solutions determined by frequency sweep test (*f* = Hz, τ = 0.07 Pa and 20°C)

Concentration/Temperature (°C)	$G^{\prime }\left(Pa\right)$	$G^{\prime \prime }\left(Pa\right)$	$\eta^{*}\left(\text{Pa}.s\right)$	Tanδ	Slope of η*–*f*
0.5%PBSG					
20	6.20 ± 0.10^g^	2.54 ± 0.05^e^	1.07 ± 0.02^g^	0.41 ± 0.00^a^	−0.67 ± 0.00^a^
60	6.63 ± 0.04^g^	2.73 ± 0.01^e^	1.14 ± 0.00^g^	0.41 ± 0.00^a^	−0.79 ± 0.00^cd^
100	8.59 ± 0.09^g^	3.07 ± 0.00^e^	1.45 ± 0.01^g^	0.36 ± 0.00^bc^	−0.77 ± 0.06^abcd^
121	10.49 ± 0.43^g^	3.51 ± 0.06^e^	1.76 ± 0.06^g^	0.34 ± 0.02^cde^	−0.69 ± 0.01^abc^
0.75%PBSG					
20	15.46 ± 1.57^f^	5.80 ± 0.46^d^	2.63 ± 0.26^f^	0.38 ± 0.01^b^	−0.68 ± 0.00^ab^
60	19.82 ± 0.35^ef^	7.02 ± 0.13^cd^	3.35 ± 0.06^ef^	0.35 ± 0.00^bc^	−0.76 ± 0.02^abcd^
100	25.19 ± 1.02^d^	7.99 ± 0.23^c^	4.21 ± 0.17^d^	0.32 ± 0.00^def^	−0.79 ± 0.05^cd^
121	22.01 ± 3.26^de^	6.85 ± 0.78^cd^	3.67 ± 0.53^de^	0.31 ± 0.01^def^	−0.76 ± 0.00^abcd^
1%PBSG					
20	40.77 ± 0.14^b^	12.66 ± 0.11^b^	6.80 ± 0.03^b^	0.31 ± 0.00^ef^	−0.78 ± 0.04^bcd^
60	34.68 ± 1.73^c^	11.92 ± 0.29^b^	5.84 ± 0.27^c^	0.34 ± 0.01^bcd^	−0.77 ± 0.04^abcd^
100	45.51 ± 0.50^b^	14.11 ± 0.15^a^	7.58 ± 0.07^b^	0.31 ± 0.01^ef^	−0.79 ± 0.02^cd^
121	51.30 ± 0.95^a^	15.23 ± 0.73^a^	8.52 ± 0.18^a^	0.30 ± 0.01^f^	−0.80 ± 0.01^d^

Note

Different letters indicate significant differences between PBSG solutions at *p* < 0.05 by Tukey test.

In general, increase in temperature from 20 to 121°C increased the *G*′ and *G*″, except at the 1% PBSG that the values of *G*′ and *G*″ in 60°C were lower than 20°C, and also at 0.75% PBSG, *G*′ and *G*″ values for 121°C were lower than 100°C. Besides, the *G*′ was constantly higher than *G*″ in all frequency ranges. This means that solutions could display a weak gel behavior and their structures are not very susceptible to temperature alterations.

The tan *δ* was in the range of 0.30–0.41 (Table 4) which specifies weak gel structure in samples. At the constant temperature, the tan *δ* value for samples with high concentration was lower indicating these samples can represent a behavior between a weak and an elastic gel (Yoshimura, Takaya, & Nishinari, 1998). Also, at the same concentration, PBSG solutions at the 121°C had the lowest tan *δ* which is related to the establishment of stronger intertwined network. Similar results were obtained for psyllium gum (Farahnaky et al., 2010) and *L. perfoliatum* gum (Hesarinejad et al., 2014).

**Figure 5:**
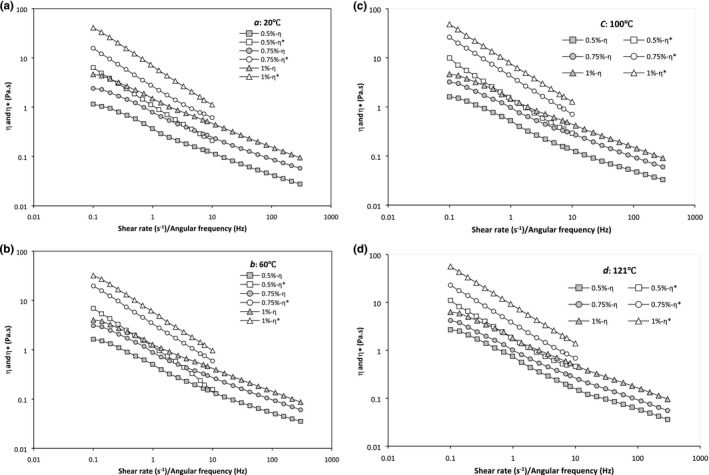
Combined plot of complex viscosity (***η*** ^*^) and apparent viscosity (***η_a_***) against angular frequency/shear rate (Cox‐Merz plot) for PBSG solutions [Correction added on 3 May 2019, after first online publication: Figure 3, 4, and 5 have been replaced with the correct images.]

The *η* ^*^ of PBSG solutions had linear decrease with frequency enhancement on a double logarithmic scale, which represents a non‐Newtonian shear‐thinning behavior (Figure 5a,b). Also, the *η* ^*^ increased with concentration enhancement from 0.5% to 1% (Table 4). The *η* ^*^ of PBSG had dependency to temperature; thus, as the temperature increases from 20 to 121°C, enhancement of *η* ^*^ was observed, except sample of 1%‐60°C that *η* ^*^ was lower than other samples, and also at 0.75%, *η* ^*^ value for 121°C was lower than that of 100°C.

The slope of *η* ^*^ against frequency (*η* ^*^−*f*) is another parameter that represents the gel strength. *η* ^*^−*f* slope near to −0.76 defines “weak gel” properties of a hydrocolloid gel organized by entangled random coil chains (Hesarinejad et al., 2014). As shown in Table 4, the *η* ^*^−*f* slope varied from −0.67 (0.5%‐20°C) to −0.80 (1%‐121°C). Thus, intertwining of PBSG chains forms a weak gel. Naji‐Tabasi and Razavi (2017b) reported that the *η* ^*^−*f* slopes for BSG fractions at 1% concentrations varied from −0.67 to −0.91.

### 3.3 Test of the Cox–Merz rule

According to Cox and Merz (1959), the *η* ^*^ and *η*_a_ parameters should be identical if gum solution do not have any energetic interactions. On the contrary, irregular biopolymers, hydrocolloid solutions with firm conformation, and regular chains show a structured liquid behavior and do not follow this law (Naji‐Tabasi & Razavi, 2017b). Figure 5 shows that the apparent viscosity, *η*_a_, in steady shear and the complex viscosity, *η* ^*^, in dynamic shear were plotted against the shear rate, $\dot{\gamma }$, and the frequency, *ω*, respectively. According to this figure, the *η* ^*^ was always higher than the *η*_a_; therefore, PBSG solutions did not obey Cox–Merz rule and exhibit gel‐like behavior, except of 0.5%‐60°C that at higher shear rate or angular velocity, *η* ^*^ became equals to *η*_a_, that is to mean, this sample followed Cox–Merz rule (Figure 5b). The deviation from Cox–Merz rule is due to various molecular rearrangements occurring in the flow patterns over the frequency range or practical shear rate (Richardson & Ross‐Murphy, 1987). As demonstrated in Figure 5a,b, the difference between *η* ^*^ and *η*_a_ increased with the increase in the concentration. Similar trend was observed with increasing temperature from 20 to 121°C. Generally, at low frequencies, in all samples, the deviation from the Cox–Merz rule was higher.

## 4 CONCLUSIONS

Herein, steady and dynamic flow characteristics of PBSG in the LVE region as a function of concentration and temperature were studied. The Herschel–Bulkley and second‐order structural kinetic models indicated well the shear dependency and time dependency of PBSG solutions, respectively. Storage (*G*′) and loss (*G*″) moduli as a function of frequency showed that the solutions revealed the rheological behavior similar to weak gel‐like macromolecular dispersions with *G*′ higher than *G*″ in all frequency ranges. Both *G*′ and *G*″ behaviors were dependent on temperature and concentration. Above results suggested that this hydrocolloid has a good potential to use in formulation of food products.

## CONFLICT OF INTEREST

The authors declare that they do not have any conflict of interest.

## ETHICAL STATEMENT

This study does not involve any human or animal testing.
